# Feasibility of Image-Guided Radiotherapy for Elderly Patients with Locally Advanced Rectal Cancer

**DOI:** 10.1371/journal.pone.0071250

**Published:** 2013-08-13

**Authors:** Nam P. Nguyen, Misty Ceizyk, Jacqueline Vock, Paul Vos, Alexander Chi, Vincent Vinh-Hung, Judy Pugh, Rihan Khan, Christina Truong, Gabby Albala, Angela Locke, Ulf Karlsson, Steve Gelumbauskas, Lexie Smith-Raymond

**Affiliations:** 1 Department of Radiation Oncology, University of Arizona, Tucson, Arizona, United States of America; 2 Department of Radiation Oncology, Lindenhofspital, Bern, Switzerland; 3 Department of Biostatistics, East Carolina University, Greenville, North Carolina, United States of America; 4 Department of Radiation Oncology, University of West Virginia, Morgantown, West Virginia, United States of America; 5 Department of Radiation Oncology, University Hospitals of Geneva, Geneva, Switzerland; 6 Department of Pathology, University of Arizona, Tucson, Arizona, United States of America; 7 Department of Radiology, University of Arizona, Tucson, Arizona, United States of America; 8 Department of Radiation Oncology, Marshfield Clinic, Marshfield, Wisconsin, United States of America; University of Wisconsin School of Medicine and Public Health, United States of America

## Abstract

**Purpose:**

The study aims to assess the tolerance of elderly patients (70 years or older) with locally advanced rectal cancers to image-guided radiotherapy (IGRT). A retrospective review of 13 elderly patients with locally advanced rectal cancer who underwent preoperative chemoradiation using IGRT was performed. Grade 3–4 acute toxicities, survival, and long-term complications were compared to 17 younger patients (<70 years) with the same disease stage.

**Results:**

Grade 3–4 hematologic toxicities occurred in 7.6% and 0% (p = 0.4) and gastrointestinal toxicities, and, in 15.2% and 5% (p = 0.5), of elderly and younger patients, respectively. Surgery was aborted in three patients, two in the elderly group and one in the younger group. One patient in the elderly group died after surgery from cardiac arrhythmia. After a median follow-up of 34 months, five patients had died, two in the elderly and three in the younger group. The 3-year survival was 90.9% and 87.5% (p = 0.7) for the elderly and younger group respectively. Two patients in the younger group developed ischemic colitis and fecal incontinence. There was no statistically significant difference in acute and late toxicities as well as survival between the two groups.

**Conclusions and Clinical Relevance:**

Elderly patients with locally advanced rectal cancers may tolerate preoperative chemoradiation with IGRT as well as younger patients. Further prospective studies should be performed to investigate the potential of IGRT for possible cure in elderly patients with locally advanced rectal cancer.

## Introduction

Standard of care for locally advanced rectal cancer has been surgery combined with chemoradiation either preoperatively or postoperatively. Preoperative chemoradiotherapy is often preferred because of improved loco-regional control and improved sphincter preservation [1]. The chemotherapy regimen is often based on 5-fluorouracil (5-FU) or capecitabine with similar efficacy when combined with radiotherapy [2]. Grade 3–4 gastrointestinal, urologic, and hematologic toxicities are frequently the limiting factors when chemotherapy is combined with three-dimensional conformal radiotherapy (3D-CRT) because of excessive irradiation of normal pelvic organs [1]–[3]. Given the fear of severe toxicity, elderly patients are often excluded from randomized trials, thus potentially depriving them from a curative treatment. Less aggressive treatment in elderly rectal cancer patients has been reported to increase their cancer-specific mortality even though they may be diagnosed in less advanced stages [4]. New modalities of radiation treatment such as intensity-modulated radiotherapy (IMRT) may potentially decrease the rates of grade 3–4 acute toxicity and improve patient tolerance to chemoradiation by generating a steep dose gradient [5]. Preliminary results with IMRT are encouraging with fewer treatment breaks, less serious toxicities, and less hospitalization in patients with rectal cancer undergoing chemoradiation with various chemotherapy regimens [6]. In addition to reduced toxicity, an excellent pathological response rate and complete resection rate are reported following chemoradiation with IMRT in patients with locally advanced rectal cancer [7]. Thus, the potential advantage in normal tissue sparing that is associated with IMRT may allow elderly patients to receive curative treatment despite the presence of age-associated co-morbidities. Image-guided radiotherapy (IGRT) is a special technique of IMRT delivery combining the steep dose-gradient with accurate daily imaging allowing for precise target radiation dose delivery and further sparing of the small bowels, bladder, and bone marrow [8], [9]. Previously, we reported the feasibility of IGRT to reduce treatment toxicity and improved pathological response in patients with locally advanced rectal cancer undergoing chemoradiation [10]. In the current study, we further assess the efficacy and toxicities of definitive radiotherapy delivered with IGRT in elderly patients receiving curative treatment for locally advanced rectal cancer.

## Materials and Methods

The medical records of 30 patients undergoing neo-adjuvant radiotherapy for locally advanced rectal cancer at the University of Arizona Radiation Oncology department were retrospectively reviewed. The University of Arizona institutional review board (IRB) approved this retrospective study and waived the requirement for patient consent because of the nature of the study. Locally advanced tumors were defined as T3, T4 tumors based on preoperative ultrasound (US) staging. All patients had a Karnofsky performance status of 70% or higher. All patients had a complete history and physical examination, a digital rectal exam, a CT scan of the chest, abdomen, and pelvis, and an endoluminal US exam. Laboratory tests included a transaminases, alkaline phosphatase, total bilirubin, and carcinoembryonic antigen. Prior to treatment, each patient was simulated in the supine position with a body vacuum bag for treatment immobilization. A computed tomography (CT) scan with and without intravenous (IV) contrast for treatment planning was performed in the treatment position. The abdomen and pelvis were scanned with a slice thickness of 3 mm. Rectal and IV contrast were employed to aid in tumor localization and in identifying grossly enlarged regional lymph node for target volume delineation. Radiotherapy planning was performed on the non-contrast enhanced CT scan to avoid possible interference of contrast density with isodose distribution calculations. Diagnostic positron emission tomography (PET)-CT scan for tumor imaging was also incorporated with CT planning when available. Normal organs at risk (OAR) for complications were outlined for treatment planning (small bowels, bladder, and femoral heads). The gross tumor volume (GTV) was outlined integrating information obtained from the CT scan with IV and rectal contrast, the endoscopic exam, and PET scanning when available. The clinical target volume (CTV) included the rectum, mesorectum, presacral space, and internal iliac nodes. The external iliac nodes were included if there was tumor extension to the vagina, uterus, cervix, prostate, or bladder. The inguinal lymph nodes were treated if there was tumor invasion of the anal canal. The planning target volume (PTV) was generated by isotropically expanding the CTV by a 1-cm margin. The peritoneal cavity was contoured to represent the small bowel volume as it seems to be the most accurate predictor of acute lower gastrointestinal toxicity to pelvic irradiation compared to contouring single bowel loops [11]. An integrated boost technique was used for the IGRT technique to treat the PTV to 45 Gy at 1.8 Gy/fraction and the GTV to 50 Gy at 2 Gy/fraction respectively. Target volume coverage was specified to be at least 95% of the prescribed dose. Dose constraints for normal organs at risk (OAR) for complications were: small-bowel volume receiving 45 Gy (V45) less than 10%, bladder: V45 less than 50%; and femoral head volumes receiving 40 Gy (V40) less than 50%. Twenty-eight patients were treated on a helical tomotherapy unit and 2 patients on a Varian EX unit. Daily MV CT (Tomotherapy) or fusion images (Varian EX) were checked for treatment accuracy. All the radiation constraints were met for tumor coverage and OAR.

### Chemotherapy

Concurrent chemotherapy was either intravenous 5-fluorouracil (5FU) or oral capecetabine. Capecetabine was given at an oral dose of 825 mg/m^2^ twice daily, 7 days weekly, beginning on the first day of radiotherapy and ending on the last day. 5-FU was administered via continuous intravenous infusion with a portable pump, at 225 mg/m^2^ daily during the whole course of radiotherapy. The patients were monitored during treatment with weekly CBC, liver enzymes, electrolytes, BUN, and creatinine. Acute and late treatment toxicity were scored according to the Radiation Therapy Oncology Group (RTOG) scale (http://ctep.cancer.gov).

### Surgery

Patients underwent surgery 6 to 8 weeks after completion of external beam radiation. All patients underwent a rectal endoscopy prior to surgery to assess tumor response following chemoradiotherapy. The type of surgical procedure was determined by the surgeon based on tumor response. A sphincter-sparing procedure was attempted if tumor shrinkage was deemed sufficient to permit complete resection with negative margins.

### Pathologic Evaluation

Resection margins were measured based from the inked surface of the surgical specimen. The circumferential resection margin (CRM) was scored as positive if the tumor was located 1 mm or less from the inked non-peritonealized surface of the specimen. A pathological complete response (pCR) was defined as no residual tumor in the surgical specimen. For patients who did not achieve a pCR, tumor size and depth of invasion were assessed on the surgical specimen and staged according to the TNM system.

### Statistical Analysis

Statistical analysis was performed with the log-rank test. A p-value of <0.05 is considered statistically significant. Survival data was analyzed using Kaplan-Meier estimation.

## Results

### Patient Characteristics

We identified 30 patients with locally advanced adenocarcinoma of the rectum treated with preoperative IGRT chemoradiation at the University of Arizona Radiation Oncology department from 2008 to 2012. Thirteen patients were 70 years or older (elderly) and 17 were less than 70 years (younger), Median age at diagnosis was 77 (range:70–85) and 62 years (range:49–68) for elderly and younger patients respectively. There were four females in both groups, nine males in the elderly group and 13 males in the younger group. All patients had T3 disease on endoscopic ultrasound. Perirectal lymph nodes were present in three patients, two of the elderly group and one of the younger group. Chemotherapy consisted of 5-FU in 19 patients (7 of the elderly and 12 of the younger group), and capecitabine in 11 patients (6 of the elderly and 5 of the younger group). Table 1 summarizes patient characteristics.

**Table 1:** Patient characteristics.

	Younger (<70)	Older (70 or older	Whole group
Patient number	17	13	30
Sex			
Male	13	9	22
Female	4	4	8
Age (years)			
Median	62	77	67
Range	49–68	70–85	49–85
Clinical stage T3	17	13	30
Chemotherapy			
5-fluorouracil	12	7	19
capecetabine	5	6	11
Follow-up (months)			
Median	43	34	42
Range	3–57	3–54	3–57

### Acute Toxicities

During radiotherapy, one patient in the elderly group developed grade 4 diarrhea secondary to pseudomembranous colitis and required a treatment break of 13 days. Another elderly cancer patient mistook capecitabine for another medication and had an overdose resulting in grade 4 diarrhea and anemia requiring repeated blood transfusion. Her radiotherapy was discontinued after 28 Gy. One patient in the younger group had grade 3 diarrhea. There was no hematologic toxicity in the younger group. One patient in the younger group developed grade 3 diarrhea.

Mean weight loss was 3.7 and 3.9 pounds for elderly and younger patients respectively. No patient in the younger group had a radiotherapy treatment break. Only one patient in the elderly group had a treatment break secondary to his pseudomembranous colitis.

Among the five patients who had chemotherapy protocol violations (dose reduction, delay or discontinuation of chemotherapy), two were in the elderly group and three in the younger group.

Among four patients who did not undergo surgery following chemoradiation, three were in the elderly group and one in the younger group. One elderly patient had surgery aborted because of liver metastases at laparotomy, another one died from cardiac arrhythmia before the scheduled surgery, and the third one declined surgery. The patient in the younger group declined surgery when she was told that she would need an abdomino-pelvic resection because of the tumor location.

All 26 operated patients achieved a complete resection with negative margins. Among the nine patients (30%) who achieved a pCR, three were in the elderly group and the other six were in the younger group.

### Treatment Outcomes

After a median follow-up of 42 months (range 3–57), the 3-year survival was estimated to be 90.9% and 87.5% for the elderly and younger patient respectively (p = 0.7). No patient who underwent surgery developed a local recurrence. The local recurrence rate was estimated to be 7.6% and 6.6% for the elderly and younger patients respectively (p = 0.4). Corresponding numbers for distant metastasis were 17.6% and 15.4% (p = 1). Among five patients who developed distant metastases (liver:4, lung and liver:1), two were in the elderly group and three were in the younger group. One patient in the younger group developed a second lung primary and was salvaged with surgery. The two patients who developed long-term complications were in the younger group. One had ischemic colitis and the other one had rectal incontinence. Table 2 summarizes treatment outcomes and toxicities in the two groups.

**Table 2:** Treatment toxicity and patient outcome.

	Younger (<70)	Older (70 or older)	p-value
Weight loss (pounds)			
Mean	3.7	3.9	0.8
Range	0–19	0–14	
Treatment breaks (days)			
Mean	0	1	0.1
Range	0	0–13	
Grade 3–4 toxicity (%)			
Hematologic	0	7.6	0.4
Gastrointestinal	5	15.2	0.5
Chemotherapy protocol violations (%)	17	15.2	0.1
Radiotherapy protocol violations (%)	0	7.6	0.4
Surgery aborted (%)	5	23	0.8
Long-term complications (%)	11	0	0.4
3-year survival (%)	90.9	87.5	0.7
Local recurrences (%)	7.6	6.6	0.4
Distant metastasis (%)	17.6	15.4	0.7

## Discussion

To our knowledge, this is the first study looking at the feasibility of IGRT for elderly patients with locally advanced rectal cancers. Despite a small number of patients, our study suggests that elderly rectal cancer patients tolerate chemoradiation quite well andare able to undergo surgery with excellent loco-regional control because of a high complete resection rate. In the past, there were concerns that elderly rectal cancer patients may not tolerate the combined modality very well because of the expected toxicity of treating a large volume of bowels with conventional chemoradiation technique [12], [13]. Indeed, in studies that reported a high rate of gastrointestinal toxicities and poor tolerance to the combined modality in elderly rectal cancer patients, one-third of the patients were treated with postoperative chemoradiation which has been associated with a higher acute and long-term toxicity compared to preoperative chemoradiation [13], [14]. In addition, most of the treatment breaks were secondary to radiation enteritis associated with excessive bowel irradiation with 3D-CRT [13]. As an illustration, death from myocardial infarction and protocol violations requiring chemotherapy dose reduction and/or radiotherapy interruption secondary to severe toxicity have been reported in 32% of patients with locally advanced cancer treated with capecitabine and oxaliplatin concurrently with 3D-CRT [15]. However, using the same chemotherapy regimen, Sola et al reported that pelvic radiation with IMRT for locally advanced rectal cancer was better tolerated with less interruption of the treatment schedule [7]. Thus, bowel sparing and bone marrow sparring through IMRTmay improve patient tolerance to chemoradiation, allowing them to undergo surgery for complete resection and possible cure for their rectal cancer [5], [6]. Image-guided radiotherapy may further improve patient tolerance to chemoradiation because of daily CT imaging allowing for more accurate radiation delivery, and rapid dose fall off comparing to conventional IMRT technique [9], [16]. A simultaneous integrated tumor boost (SIB) delivering a higher radiation dose to the gross tumor while sparing the normal organs may also be achieved to improve local control without excessive increase in acute toxicity [17]. The favorable acute toxicity profile of IGRT may be beneficial to improve elderly rectal cancer patients tolerance to radiation and allow them to have curative resection despite the associated co-morbidity. Indeed, in our study, all patients tolerated chemoradiation quite well and elderly patients fared as well as younger patients with no significant difference in treatment break or weight loss. Acute grade 3–4 toxicity was acceptable in both groups. Our results compared favorably with other studies using IMRT with chemotherapy for locally advanced rectal cancer. The reported acute grade 3–4 radiation enteritis and hematologic toxicity range from 3% to 24% and 0 to 3% respectively. The percentage of patients who have radiotherapy treatment break range from 11.1% to 16.2% [7], [18]–[20]. Table 3 summarizes acute toxicity in patients with locally advanced rectal cancer treated with IMRT and chemotherapy.

**Table 3:** Treatment toxicity reported in studies using IMRT and chemotherapy for the treatment of locally advanced rectal cancer.

Study	Patient No	Grade 3–4 gastrointestinaltoxicity (%)	Grade 3–4 hematologictoxicity (%)	Treatment breaks (%)
Arbea et al [7]	100	21	2	14
Samuelan et al [15]	31	3	3	16.2
Li et al [16]	63	9.5	1.6	11.1
Ballanoff et al [17]	8	12	0	12

Despite the small number of patients, our study highlights the possibility that elderly rectal cancer patients may tolerate chemoradiation better with new techniques of radiotherapy that spare the normal pelvic organs from excessive radiation. Most often, elderly rectal cancer patients are deprived from chemotherapy or radiotherapy because of the age bias [21], [22]. The nihilistic attitude toward elderly patients results in sub-optimal treatment and poor survival (22). Elderly rectal cancer patients who were able to undergo surgery had a significantly better survival compared to the ones who did not have surgery [23]. Image-guided radiotherapy may provide elderly cancer patients a better chance for curative resection and improved survival.

The limitations of the present study include its retrospective nature, the small number of patients, the absence of co-morbidity information, and the relatively short median follow-up. Nevertheless, IGRT may be a new treatment modality to reduce acute toxicity during chemoradiation for locally advanced rectal cancer and improve tolerance to treatment in elderly cancer patients. Further studies with a larger patient population should be performed to investigate the potential of IGRT for curative treatment in elderly rectal cancer patients.

### Conclusion

Image-guided radiotherapy in the setting of chemoradiation is well tolerated in elderly patients with rectal cancer. The potential of IGRT to help elderly rectal cancer patients achieve a curative resection should be investigated in future clinical trials.
